# Letters to the Editor

**Published:** 1987-11

**Authors:** Tim Harlow

**Affiliations:** The Health Centre Cullompton Devon


### Letters to the Editor

### AIDS Epidemic and Control.

Sir,

I thank Dr. John Seale for his stimulating article on AIDS Epidemic and Control, your Journal 102 (iii) August '87 pages 66–68. I must agree how important an accurate understanding of the disease is for our profession and for the public. However, there are a number of points which I feel bound to question, some of which it would be wrong to allow to pass unchallenged.

Several extrapolations were made from animal diseases and from different human disease which implied that AIDS was likely to be spread by inhalation, casual social contact and insects. Despite the initial plausibility of these concepts the fact remains that AIDS has not been recorded to spread by means other than blood, via breaks in the skin or mucous membranes or by penetrative sexual activity. Household contacts of AIDS patients and people positive for the HIV antibody do not seroconvert. The virus being viable after long periods in water at room temperature cannot be taken to mean that it is a significant danger of infection under those circumstances if the recognised methods for the control of infection by the AIDS virus are followed. The mere presence of the virus does not imply that it can lock on to the T4 receptor sites on cell membranes and therefore infect. A study of a 140 prison officers bitten by AIDS patients have shown none to seroconvert. Dr. Seale's interesting theory of homosexual conspiracy is not supported with the documented change in homosexual behaviour with drastic reduction in promiscuity and the overall responsibility to the community at large demonstrated by the majority of the homosexual population in the control of AIDS.

It is difficult to explain the incidence of HIV in Central Africa (15% of heterosexuals) on the principle Dr. Seale suggests about homosexuality being a uniquely efficient method of transmission of the virus.

Compulsory testing is a matter for which a case can be argued either way. I do not propose to discuss this in detail here but it would be interesting to know what Dr. Seale suggests the government do with those people identified as HIV carriers. Dr. Seale is right to stress the difficulty with developing vaccines or with treatments for AIDS and to emphasise the need for prevention. However, there is a danger of muddled thinking in such an emotive subject and as doctors we must not allow our views to be shaped by other than established fact and logic.

Yours sincerely,
Dr Tim Harlow
The Health Centre
Cullompton
Devon

